# Effects of dietary β-glucan and rice fermented on growth performance, fatty acids, and Newcastle disease immune response in turkey broilers

**DOI:** 10.1016/j.sjbs.2023.103736

**Published:** 2023-07-13

**Authors:** Nguyen Hoang Qui, Nguyen Thuy Linh

**Affiliations:** Department of Animal Science and Veterinary Medicine, School of Agriculture and Aquaculture, Tra Vinh University, Tra Vinh Province, Vietnam

**Keywords:** Fermented feed, β-glucan, Fatty acid, Immunity, *Saccharomyces cerevisiae*, Newcastle disease

## Abstract

Poultry production has been developing in Vietnam with challenges of disease. Thus, feed additive should be investigated not only growth but also health enhancement. Here, we aimed to determine the effects of *Saccharomyces cerevisiae*-fermented rice (FR) and β-glucan on turkey’s growth performance, carcass characteristics, immune and fatty acid (FA) profiles. A total of 180 turkey chicks aged 1–56 days were randomly assigned to five sextuplicate groups and the birds had *ad libitum* feed and water access throughout the experiment. The five treatment groups were given the same diet with different proportions of FR and β-glucan. Broilers supplemented with 4% β-glucan and 4% FR presented the highest and second-highest growth performance, respectively. The 4% β-glucan and 4% FR treatments resulted in the highest carcass characteristic values without significantly affecting the breast or thigh meat pH or cooking loss. The 4% β-glucan and 4% FR treatments maximally increased the Newcastle disease (ND) antibody titers at 28, 42 and 56 days, respectively as well as thymus organ index. The foregoing treatments did not significantly affect the blood profiles relative to the control. However, the 4% FR treatment lowered the blood cholesterol levels (p > 0.05). The total FA profiles did not significantly differ among treatments. Nevertheless, both the β-glucan and FR treatments increased the MUFA levels compared to that of the control (p > 0.05). Hence, the dietary administration of 4% β-glucan and FR to turkey broilers could effectively improve their growth performance and immunity.

## 1 Introduction

The poultry industry of Vietnam has expanded in recent years. However, progress in poultry husbandry has been impeded by various obstacles including infections. Newcastle disease is caused by a viral pathogen and has become widespread in global poultry husbandry. It can lead to low growth performance and high mortality and adversely affects productivity and economic efficiency in poultry production (Karthikeyan et al., 2020). Newcastle disease are one of the most prevalent poultry diseases in Vietnam, particularly in the Mekong delta (Delabouglise et al. 2020). A recent study of Carrique-Mas et al. (2019) found that 2.5% of poultry in small-scale flocks in the region died each week on average. Moreover, restriction of the use of antibiotics in feed has hindered poultry growth and health performance as there are currently few available prophylactic or therapeutic alternatives for them.

Animal feed has been supplemented with herbs to improve performance and immunity. Furthermore, the integration of fermented rice (FR) and other similar ingredients into fodder has become a research hotspot. Prior studies demonstrated that fermented feed improves intestinal morphology, boosts immunological responses, reduces oxidative stress, and alters the microbial community in the gut of broilers (Yan et al., 2019) and layers (Zhu et al., 2020). Fermentation also increases the nutritional value of rice. FR extracts prevent NaCl or methotrexate cytotoxicity in the gastric mucosa (Ochiai et al., 2013), enhance antioxidant activity (Shin et al., 2019, Xu et al., 2019), have anti-inflammatory efficacy in the intestinal mucosa (Oh et al., 2020), lower blood and hepatic cholesterol levels, and exhibit anti-steatosis activity (Bunnoy et al., 2015). Rice extracts fermented with *Aspergillus oryzae* and/or *Saccharomyces cerevisiae* (*S. cerevisiae*) may be administered as anti-influenza therapy and are sources of anti-influenza drugs (Shoji et al., 2021). *S. cerevisiae*-based fermentation contains abundant β-glucans which improve intestinal health and increase post-vaccination antibody titers in poultry (Dewi et al., 2021). Thus, FR should also improve growth performance. Horst et al. (2019) stated that dietary β-glucan can improve the host immunological response to Newcastle vaccinations. Furthermore, β-glucan extracted from yeast increases the number of cells releasing immunoglobulin A (IgA) and could, therefore, substitute for antibiotics in the treatment of certain enteric pathogens in poultry (Anwar et al., 2017). *S. cerevisiae* can also increase the phosphorus and crude protein and decrease the fiber content in the feed. Several fungi including *S. cerevisiae* can detoxify feed (Hassaan et al., 2015), reduce the levels of phytate and other antinutritional substances in it, and enhance the bioavailability of the nutrients in it. FR (Jayachandran et al., 2018) and β-glucan improve gastrointestinal tract capacity and microbial activity (Azrinnahar et al., 2021). Linh et al. (2021) and Azrinnahar et al. (2021) mentioned that the growth performance of chickens administered *S. cerevisiae-*fermented feed was superior to that of the birds fed a control diet. Besides, Linh et al. (2021) mentioned that 4% of fermented rice by *S. cerevisiae* maximally increased growth performance of local chicken effectively, but no further information was provided.

Hence, the provision of *S. cerevisiae*-fermented rice to poultry is especially important when the incorporation of antibiotics into the feed is restricted. However, the effects of *S. cerevisiae*-fermented rice on the immune response to Newcastle disease and the FA profiles of poultry are unknown. Thus, the present study aimed to establish the impact of *S. cerevisiae*-fermented rice and β-glucan on the growth performance, meat quality, blood biochemical profiles, and immune response in turkey broilers.

## 2 Materials and methods

### 2.1 Study site and animal ethics

The present study was conducted on the experimental farm of Tra Vinh University, Tra Vinh City, Vietnam (9°55′05.8″N and 106°21′00.3″E) between December 2022 and February 2023 following the methodological recommendations of the Committee of Education and Science of Tra Vinh University and under approval No. 137/2022/HĐ.HĐKH&ĐT-ĐHTV.

### 2.2 Experimental design and diet

The trial was a completely randomized design. One hundred eighty chicks (Bronze turkey strain) were randomly assigned in lots of six to five different sextuplicate experimental groups. All birds were housed under hygienic conditions in 1.5 m × 1.5 m cages as prescribed for turkey fattening. An 18 L:6 D lighting program was in use. After being kept constant at 34 °C for five days, the temperature in the room was reduced by 2 °C every week until it approximately reached 26 °C, where it remained until the end of experiment. All broilers had *ad libitum* food and water access. Rice husks and Balasa bio-yeast (Minh Tuan factory, Ha Noi, Vietnam) were spread around the cage floors. All cages were cleaned weekly to maintain hygiene. The crude protein, dry matter, organic matter, total mineral, calcium, and phosphate content was measured for all feed ingredients before blending (Table 1). The experimental diet was formulated for birds in the age ranges of 1–28 days and 29–56 days (NRC, 1994) (Table 2). The feed combinations were prepared once weekly, consisted of locally sourced corn, broken rice, bran, soybean meal, fish meal, dicalcium phosphate, stone meal, lysine, methionine, mineral premix, and salt, and were stored in a cold, dark place (Table 3). The birds in Group I (CTR) received basal diet without FR or β-glucan. Those in Groups II (FR1), III (FR2), IV (FR3), and V (GLU) received basal diet with 4% aqueous extract, 4% solid extract of FR mixture, and 4% fully fermented rice, respectively. Those in Group V (GLU) received feed with 4% of 40,000 mg/kg β-glucan (Dat Viet Co., Vietnam).

**Table 1:** The chemical compositions of ingredients in the diets (% DM).

Ingredients	CP	DM	EE	NFE	OM	CF	Ca	P	ME (Kcal/Kg DM)
Broken rice	7.98	86.2	0.91	90.7	99.7	0.10	0.020	0.100	3488
Fish meal	63.7	91.6	10.0	11.7	85.8	0.40	3.300	2.430	3223
Corn	7.15	87.2	1.80	88.2	98.4	1.24	0.004	0.140	3699
Soybean meal	45.5	87.2	1.73	43.3	94.2	3.70	0.250	0.640	2661
Bran	13.2	88.7	8.25	63.6	92.6	7.60	0.030	2.030	2608
DCP	–	100	–	–	14.8	–	22.0	17.00	–
Stone meal	–	100	–	–	–	–	37.9	0.01	–
Methionine	–	99.3	–	–	–	–	–	–	–
Lysine	–	97.4	–	–	–	–	–	–	–
Mineral-premix	–	100	–	–	–	–	–	–	–

CP: crude protein, ME: metabolizable energy, DM: dry matter, OM: organic matter, EE: ether extract, NFE: nitrogen-free extract, CF: crude fiber, Ca: calcium, P: phosphate.

**Table 2:** The ingredient compositions in the experimental diets (%).

**Ingredients**	**Growth periods**	
	**1**–**28 days old**	**29**–**56 days old**
Broken rice	13.0	12.5
Bran	29.0	28.3
Corn	24.0	30.2
Fish meal	8.0	7.6
Soybean meal	22.8	18.0
Stone meal	2.00	2.20
Dicalcium phosphate	0.3	0.3
Lysine	0.20	0.20
Methionine	0.10	0.10
Mineral-premix*	0.30	0.30
Salt	0.30	0.30
Total	100	100

*: Mineral premix was mixed according to Vitamin A: 2,500,000 UI; Vitamin E: 4,000 mg; Vitamin K3: 400 mg; Vitamin D3: 600,000 UI; Choline: 100,000 mg; Mangan: 14 g; Folic acid: 80 mg; Cu: 48 g; Fe: 32 g; Zn: 40 g; Se: 0.04 g; Iodine: 0.5 g; Co: 0.28 g.

**Table 3:** The proximate analysis of experimental feed.

**Proximate analysis**	**Growth periods**	
	**1**–**28 days old**	**29**–**56 days old**
CP (%)	22.0	20.0
ME (Kcal/kg DM)	2961	3009
P (%) Ca (%)	1.04 1.28	1.00 1.34
NDF (%)	15.9	16.1
NEF (%)	62.1	64.3
Lys (%)	1.27	1.18
Met (%)	0.55	0.52
Ash (%)	5.35	5.08
EE (%)	4.18	4.10
CF (%)	3.39	3.24
ADF (%)	6.31	6.01

CP: crude protein, ME: metabolizable energy, DM: dry matter, OM: organic matter, EE: ether extract, NFE: nitrogen-free extract, NDF: neutral detergent fiber, CF: crude fiber, Ca: calcium, P: phosphate, Lys: lysine, Met: methionine, ADF: acid-detergent fiber.

All birds were administered Newcastle disease vaccine at 3 days followed by a booster at 14 days. The vaccines included strain F which is highly prevalent in Vietnam. Gumboro and Avian influenza vaccines were administered on days 7 and 16, respectively.

### 2.3 Fermented rice preparation

Dehusked rice (1.5 kg) was placed in a can containing 1.5 L water and the mixture was stored at room temperature at approximately 27 °C for 5 h. Then, 0.5 g *S. cerevisiae* (1.9 × 10^10^ CFU/g; IC Food Co. Ltd., Daejeon, Korea) was added to the mixture and the latter was stirred until fully homogenized. The mixture was maintained at room temperature in the dark for 7 days. After the container was opened, the mixture was homogenized and passed through a cloth to separate the liquid and solid fractions. The fermented mixture was only used for 3 days to minimize oxidation. The solid and liquid fractions comprised 59% and 41% of the total weight, respectively as similar to the study of Linh et al. (2021). The pH and chemical compositions of the fermented mixture are shown as follows pH was 4.0, DM of 60.32%, CP of 3.38%, EE of 0.82%, CF of 0.14%, ME of 162 Kcal/kg, and carbohydrate of 35.48%. The amount of β-glucan in fermented rice is less than 1%.

### 2.4 Growth performance

Each bird was weighed on the first day of the experiment. The birds were fed in the morning and again throughout the day. The birds were reweighed weekly and before each meal and changes in body weight (BW) were calculated and recorded. Based on the amount of food ingested and the number of orts, the daily feed intake was calculated and recorded before the morning feeding. Daily death tolls were recorded (no death during experiment time), and the feed conversion ratio (FCR) was calculated from the quotient of the feed intake (FI) (g) and the body weight gain (BWG) (g). After the 56-day rearing period, two birds per experimental group were euthanized. All birds were weighed and certain individuals (60 birds) were selected for analysis depending on their mean body weight (average of 1150 ± 150 g). On the day the birds were slaughtered, their corpses were de-feathered and eviscerated, their internal organs were measured, and their carcass characteristics and meat quality were evaluated. The carcasses, thighs, breasts, livers, gizzards, spleens, bursae of Fabricius, and thymuses were weighed. The immune organ indices were calculated as shown in Eq. (1):

(1) 1000×(immuneorganweight[g]/BW[g])

The thigh and breast flesh pH were measured with a digital pH meter (pH/ORP/Temperature Laboratory Bench Meter Mi 151; Milwaukee Instruments, Inc., Rocky Mount, NC, USA). The pH meter was cleaned and calibrated according to the manufacturer’s instructions. Thigh and breast meat samples (after 45 min of slaughter) were collected from each experimental unit, weighed, cooked in clean and fresh water for 5 min, and reweighed to determine the cooking loss (final – initial weight). The remainder of the breast meat was then subjected to FA profile analysis. The cooking loss was showed in Eq.2:

(2) $$Cooking\;loss\;\left(\%\right)\;=\;\left(\frac{final\;weight\;-\;initial\;weight}{initial\;weight}\right)\;\times \;100$$

### 2.5 Blood profiles

Total cholesterol, albumin, total protein, low-density lipoprotein cholesterol (LDL-c), high-density lipoprotein cholesterol (HDL-c), globulin, and triglycerides were measured and recorded in mg/dL. Two birds aged 56 d were randomly selected from each experimental unit. At the end of the trial, ∼2 mL of blood was drawn from each bird by using 5-mL disposable syringes fitted with 23-g needles. The blood samples were immediately placed in hematological tubes containing anticoagulant EDTA and stored in a cold bag within 48 h sample collection. The blood samples were then delivered to an animal hospital and subjected to biochemical and hematological analyses in a Cobas 6000 Analyzer (Roche Diagnostics, Basel, Switzerland).

### 2.6 Antibody titer

Before doing ND vaccine, birds were checked antibody titer to ascertain negative to ND. Two to five milliliters of blood were drawn from the wing veins of birds aged 28, 42, and 56 days according to Vietnamese protocol No. QCVN 01–83:2011/BNNPTNT. The blood was drawn into prelabeled syringes and the air therein was purged. The syringes were set on their sides in a sample container and left to coagulate at room temperature in the dark for 1–2 h. The syringes were transported in a storage container at 2–8 °C to a sample analysis center where the Newcastle virus antibody titers in the blood were determined using an enzyme-linked immunosorbent assay (ELISA) kit (IDvet; Innovative Diagnostics, Grabels, France). Each well in the kit received 100 µL serum. Post-vaccination antibody titers ≥993 indicated active protection.

### 2.7 Fatty acid profiles

The fats in the tissue samples were converted to fatty acid methyl esters according to AOAC Method No. 996.06 and the saturated, unsaturated, polyunsaturated, and monounsaturated FA compositions were detected and quantitated by gas chromatography (Agilent Technologies, Santa Clara, CA, USA). The GC had a split-splitless injector, a flame ionization detector (FID), and a 30-m fused silica capillary column. The carrier gas was helium, and the injector and detector temperatures were 250 °C and 300 °C, respectively.

### 2.8 Data analysis

All data were initially processed in Microsoft Excel 365 (Microsoft Corp., Redmond, WA, USA) and using a generalized linear model (GLM) in Minitab v. 2016 (Minitab, LLC, State College, PA, USA). GraphPad Prism v. 9 (GraphPad Software, La Jolla, CA, USA) was used to plot the graphs. Tukey’s test was used to compare treatment means. p < 0.05 indicated statistical significance.

## 3 Results

### 3.1 Turkey broiler growth performance

The FR supplement had a positive impact on all groups (Table 4). The initial weights did not influence the outcome as they did not significantly differ among treatments. Dietary FR3 supplementation increased the final weight body and the BWG, decreased FI and improved the feed conversion ratio (FCR) (p < 0.05). The highest BW and BWG occurred in the GLU treatment followed by the FR3 treatment. The highest amounts of feed consumed were recorded for the CTR, F1, and F2 treatments (p < 0.05). Dietary GLU and FR3 supplementation improved FCR to a greater extent than the other treatments except at the 28–56-day growth phase. A comparison of the total BWG, FI, and FCR values among treatments indicated that 4% β-glucan and 4% FR supplementation were optimal under the conditions of the present study.

**Table 4:** The effects of FR and β-glucan on growth performance.

**Criteria**	**Treatments**					**SEM**	***p-*value**
	**CTR**	**FR1**	**FR2**	**FR3**	**GLU**		
BW (g/bird)							
Initial weight	52.80	53.06	52.83	52.81	52.87	0.154	0.749
7 days old	89.03^c^	92.70b,c	94.73b,c	98.07^b^	106.1^a^	1.513	0.001
28 days old	373.2^c^	378.6^c^	388.2b,c	407.3^b^	440.3^a^	4.648	0.001
56 days old	1084^d^	1141^c^	1153^c^	1235^b^	1281^a^	9.802	0.001
BWG (g/bird/day)							
1–28 days old	11.45^c^	11.63^c^	11.98^b,c^	12.66^b^	13.84^a^	0.166	0.001
28–56 days old	25.40^c^	27.24^b^	27.34^b^	29.59^a^	30.02^a^	0.325	0.001
1–56 days old	18.42^d^	19.42^c^	19.66^c^	21.12^b^	21.93^a^	0.175	0.001
FI (g/bird)							
1–28 days old	22.28^a^	21.51a,b	20.70b,c	20.11 c,d	19.68^d^	0.220	0.001
28–56 days old	74.47^a^	72.75^a^	71.81^a^	61.43^b^	64.09^c^	0.683	0.001
1–56 days old	48.38^a^	47.13a,b	46.26^b^	41.18^c^	41.89^d^	0.391	0.001
FCR							
1–28 days old	1.913^a^	1.807^a^	1.659^b^	1.551^b^	1.378^c^	0.027	0.001
28–56 days old	3.221	2.936	2.948	3.305	2.888	0.116	0.061
1–56 days old	2.567^a^	2.372^ab^	2.303a,b	2.428b,c	2.133^c^	0.055	0.001

a,b,c,d : Mean values with different letters are statistically significant at p < 0.05. BW: body weight, BWG: body weight gain, FI: feed intake, FCR: feed conversion ratio, SEM – standard error of the mean, CTR: control, FR: fermented rice.

### 3.2 Turkey broiler carcass characteristics

The carcass characteristics of the birds in all treatments were superior to those of the control turkeys (Table 5). The carcass weight was highest for GLU and FR3 (p < 0.05), the breast and thigh weights were highest for GLU (p < 0.05), and the carcass, breast, and thigh percentages did not significantly differ among treatments. The heart weights did not significantly differ among treatments (p > 0.05) while liver and gizzard showed a significant difference (p < 0.05). FR3 and GLU supplementation improved the performance of the carcass meat but not those of the internal organs.

**Table 5:** The effects of FR and β-glucan on carcass characteristics.

**Criteria**	**Treatments**					**SEM**	***p*-value**
	**CTR**	**FR1**	**FR2**	**FR3**	**GLU**		
Live weight, g/bird	1028^c^	1051^c^	1131^b^	1177^a^	1214^a^	9.791	0.001
Carcass weight, g/bird	646.1^c^	661.5^c^	711.8^b^	739.2^ab^	768.5^a^	9.013	0.001
Carcass percentage, %	62.83	62.98	62.90	62.76	63.29	0.728	0.987
Breast weight, g/bird	124.6^c^	127.4^bc^	131.4^b^	140.4^a^	142.6^a^	1.257	0.001
Breast percentage, %	19.31	19.29	18.46	19.01	18.56	0.263	0.088
Thigh weight, g/bird	121.1^c^	126.0^bc^	132.4^ab^	135.4^ab^	141.6^a^	2.493	0.001
Thigh percentage, %	18.73	19.07	18.61	18.34	18.42	0.307	0.488
Liver weight, g/bird	21.70^c^	22.77^bc^	25.43^abc^	26.17^ab^	24.70^a^	0.798	0.003
Heart weight, g/bird	5.100	4.800	5.100	5.130	5.770	0.246	0.116
Gizzard weight, g/bird	34.83^b^	38.33^b^	32.80^ab^	32.73^ab^	34.33^a^	1.194	0.018

a,b,c : Mean values with different letters are statistically significant at p < 0.05. SEM – standard error of the mean, CTR: control, FR: fermented rice.

### 3.3 Turkey broiler carcass quality

There were no significant differences among treatment groups in terms of carcass quality including breast and thigh meat pH and cooking weights (Table 6). The supplements resulted in higher meat pH than the control diet, however, the difference was not statistically significant (p > 0.05). The cooked breast meat from the birds under FR3 and the cooked thigh meat from the turkeys under GLU retained more water and showed less cooking loss than the breast and thigh meat from the birds in the control group.

**Table 6:** The effects of FR and β-glucan on carcass quality.

**Criteria**	**Treatments**					**SEM**	***p*-value**
	**CTR**	**FR1**	**FR2**	**FR3**	**GLU**		
pH							
Breast	6.163	6.310	6.157	6.257	6.310	0.062	0.243
Thigh	6.820	6.890	6.897	6.870	6.943	0.083	0.881
Cooking weight (g)							
Breast							
Before cooking	5.667	5.750	5.833	5.883	5.733	0.058	0.408
After cooking	4.033	4.117	4.133	4.108	4.117	0.092	0.947
Thigh							
Before cooking	5.133	5.150	5.100	5.133	5.100	0.083	0.990
After cooking	3.667	3.683	3.683	3.685	3.692	0.052	0.998
Cooking loss (%)							
Breast	28.76	28.41	29.17	28.31	28.38	1.587	0.995
Thigh	28.39	28.41	27.74	27.99	27.54	1.725	0.995

SEM – standard error of the mean, CTR: control, FR: fermented rice.

### 3.4 Turkey immune organ indices

**Fig. 1:**
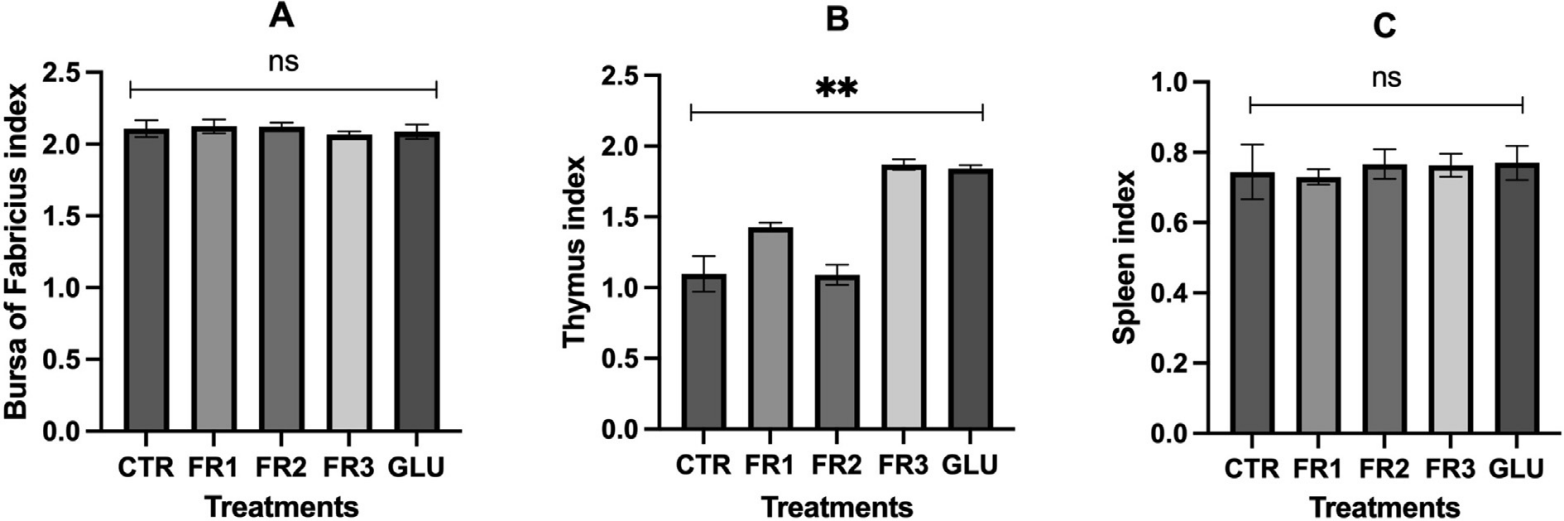
The effects of FR by *S. cerevisiae* and β-glucan on immune organ indices at 56 days. A: Effects of fermented rice by *S. cerevisiae* and β-glucan on Fabricius index. B: Effects of fermented rice by *S. cerevisiae* and β-glucan on thymus index (p < 0.05) with the highest result in treatment of FR3. C: Effects of fermented rice by *S. cerevisiae* and β-glucan on spleen index (p > 0.05). *CTR: control, FR: fermented rice.*

FR and GLU supplementation positively affected the immune organ indices (Fig. 1). The birds in the FR3 and GLU groups had the highest and second highest thymus indices, respectively, relative to the control (p < 0.05). The turkeys under the GLU and FR2 treatments had the highest and second highest spleen indices, respectively compared to the control (range: 0.730–0.770). The bursa of Fabricius indices were in the range of 2.086–2.123 and neither FR nor GLU supplementation increased them.

### 3.5 Turkey antibody titers

**Fig. 2:**
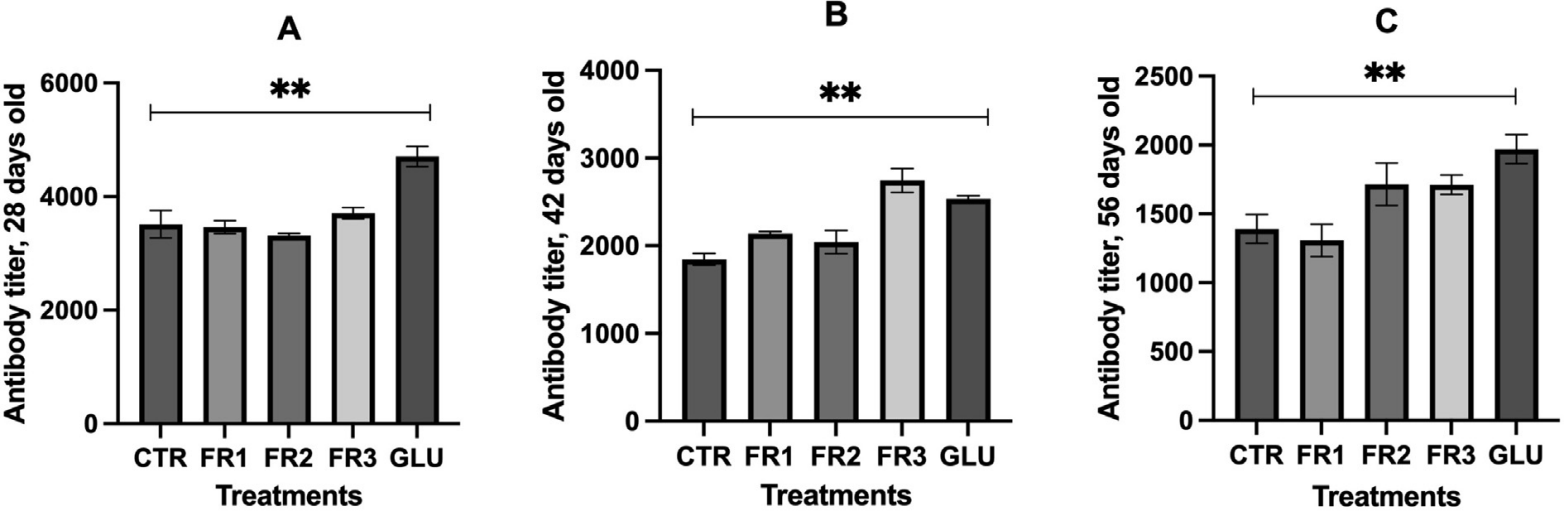
Antibody titres in case of immune response to Newcastle disease virus by FR by *S. cerevisiae* and β-glucan. A: Effects of fermented rice by *S. cerevisiae* and β-glucan on antibody titres at 28 days old. B: Effects of fermented rice by *S. cerevisiae* and β-glucan on antibody titres at 42 days. C: Effects of fermented rice by *S. cerevisiae* and β-glucan on antibody titres at 56 days. *CTR: control, FR: fermented rice.*

The mean antibody titer gradually decreased from 4707 (28 days) to 1970 (56 days) (Fig. 2). GLU supplementation increased the antibody titer. At 28 days, the antibody titers were 4707 and 3514 under GLU and the control, respectively (p < 0.05). At 42 days, the antibody titers were 2746 and ∼ 1840 under FR3 and the control, respectively (p < 0.05). Though the FR2, FR3, and GLU treatments resulted in higher antibody titers than the control diet at 56 days, the differences among groups were also significant (p < 0.05).

### 3.6 Blood lipid profiles of 56-day turkeys

There were no significant differences among treatments (Table 7) in terms of their relative impact on total protein, albumin, globulin, triglycerides, cholesterol, HDL-c, or LDL-c (p > 0.05). The birds under the GLU treatment presented with the highest protein and globulin levels while those in the FR3 groups exhibited the lowest total cholesterol levels. However, the differences among treatments were not significant.

**Table 7:** The effects of FR and β-glucan on blood lipid profiles.

**Criteria**	**Treatments**					**SEM**	***p*-value**
	**CTR**	**FR1**	**FR2**	**FR3**	**GLU**		
Total protein, mg/dL	3370	3743	3690	3870	3870	104.9	0.267
Albumin, mg/dL	1273	1422	1406	1385	1385	34.39	0.379
Globulin, mg/dL	2093	2316	2280	2483	2486	83.28	0.264
Total cholesterol, mg/dL	14.40	16.14	18.30	10.98	23.94	2.468	0.177
Triglycerides, mg/dL	85.02	89.88	97.98	93.30	79.44	5.488	0.617
LDL-cholesterol, mg/dL	51.78	51.60	58.56	59.70	44.10	3.380	0.307
HDL-cholesterol, mg/dL	26.70	30.96	31.14	28.62	27.18	2.999	0.753

SEM – standard error of the mean, CTR: control, FR: fermented rice.

### 3.7 Fatty acid profiles of 56-day turkeys

Linoleic acid (C18:2n-6) and palmitic acid (C16:0) had the highest concentrations in the turkey breast muscle followed by oleic acid (C18:1n-9), stearic acid (C18:0), and palmitoleic acid (C16:1) in descending order of concentration. The breast muscle saturated fatty acid (SFA) content was in the range of 33.47–34.08. The birds under the GLU and FR treatments displayed no different data in total breast SFA (p > 0.05). There were no significant differences among groups in terms of total breast muscle MUFA or PUFA content. The effects of FR and GLU significantly changed the content of some FAs in breast muscle, however, the amount of changing is not enough for increased or decreased the contents of total SFA/UFA in breast (Table 8).

**Table 8:** The effects of FR and β-glucan on fatty acid profiles.

**Criteria**	**Treatments**					**SEM**	***p*-value**
	**CTR**	**FR1**	**FR2**	**FR3**	**GLU**		
C12:0 (lauric acid)	0.222^a^	0.171^ab^	0.162^ab^	0.103^b^	0.165^ab^	0.020	0.010
C14:0 (myristic acid)	0.952	0.893	0.881	0.863	0.788	0.063	0.495
C14:1 (myristoleic acid)	0.120^ab^	0.145^a^	0.142^ab^	0.120^ab^	0.106^b^	0.008	0.022
C15:0 (pentadecanoic)	0.154^b^	0.148^b^	0.140^b^	0.145^b^	0.171^a^	0.003	0.000
C16:0 (palmitic acid)	25.15	25.31	25.50	24.97	25.12	0.409	0.912
C16:1 (palmitoleic acid)	3.760^b^	4.810^ab^	5.062^a^	4.990^a^	4.063^ab^	0.274	0.006
C17:0 (margaric acid)	0.231^ab^	0.210^c^	0.194^bc^	0.217^abc^	0.241^a^	0.007	0.001
C18:0 (stearic acid)	7.043^a^	6.492^b^	6.473^b^	6.893^ab^	6.748^ab^	0.175	0.003
C18:1 (elaidic acid)	0.138	0.148	0.147	0.146	0.151	0.003	0.096
C18:1n9 (oleic acid)	22.33	21.40	21.14	21.41	22.86	0.577	0.089
C18:2n6 (linoleic acid)	37.33	37.91	37.91	37.88	37.06	0.263	0.203
C18:3 (alpha-linolenic acid)	0.806	0.775	0.793	0.803	0.780	0.022	0.819
C18:3 (gamma-linolenic acid)	0.060	0.054	0.054	0.051	0.053	0.002	0.247
C20:0 (arachidic acid)	0.224^b^	0.220^b^	0.216^b^	0.217^b^	0.296^a^	0.016	0.001
C20:1 (eicosenoic acid)	0.337^b^	0.325^b^	0.357^ab^	0.339^b^	0.379^a^	0.008	0.002
C20:2n6 (Eicosadien)	0.123^a^	0.097^ab^	0.094^b^	0.102^ab^	0.117^ab^	0.006	0.018
C20:3n6 (Eicosatri)	0.099	0.086	0.082	0.092	0.099	0.005	0.164
C20:4 (arachidonic acid)	0.584	0.479	0.382	0.363	0.468	0.059	0.102
C20:5n3 (Eicosapentaenoic acid)	0.047	0.046	0.041	0.044	0.040	0.003	0.431
C22:0 (Behenic acid)	0.055	0.039	0.032	0.038	0.045	0.005	0.060
C22:6 (Docosahexaenoic acid)	0.169	0.173	0.160	0.155	0.178	0.013	0.716
C24:0 (Lignoceric acid)	0.047^b^	0.040^ab^	0.031^b^	0.047^ab^	0.049^a^	0.002	0.000
Saturated fat acid	34.08	33.52	33.63	33.47	33.62	0.390	0.822
Unsaturated fatty acid	65.92	66.47	66.36	66.52	66.36	0.527	0.932
PUFA	39.22	39.63	39.51	39.50	38.80	0.217	0.082
MUFA	26.69	26.85	26.86	27.02	27.57	0.393	0.573

a,b,c : Mean values with different letters are statistically significant at p < 0.05; SFA: Saturated fat acid; USFA: Unsaturated fatty acid; PUFA: polyunsaturated fatty acid; MUFA: monounsaturated fatty acid, CTR: control, FR: fermented rice.

## 4 Discussion

The present study revealed that β-glucan and FR supplementation improved growth performance in turkey broilers. Though the former was relatively more efficacious, the latter could be an acceptable alternative. Amer et al. (2022) reported that β-glucan was as effective as virginiamycin at improving growth performance. Chen et al. (2019) proposed that β-glucan strengthens the intestinal barrier and maintains mucous membrane integrity by inducing neurotransmitters associated with crosslinked protein, serotonin phosphate, and acetylcholinesterase in the intestinal epithelium. Moreover, it augments gastrointestinal tract functionality by stimulating intestinal peristalsis (motility) and promotes the diversity and richness of the intestinal microbiota by recruiting it for fermentation (Jayachandran et al., 2018). Dietary β-glucans supplementation might alter the microbiota, increase the abundance of beneficial microorganisms, and depopulate pathogens in the gut. Here, β-glucans were fodder for the microbiota in the lower gastrointestinal tract of the broilers. The β-glucans influenced microbial community composition and metabolic output and promoted short-chain fatty acid fermentation. The increase in nutrient content resulting from rice fermentation could account for the observed improvements in broiler growth performance in response to FR supplementation. Azrinnahar et al. (2021) stated that during fermentation, yeasts secrete enzymes that enhance nutrient availability. The inclusion of fermented products in the diet improved feed consumption efficiency in chickens (Azrinnahar et al., 2021). Microbial abundance increases in fermentation media and the bacteria serve as probiotics. As the rice was fermented by *S. cerevisiae*, β-glucans with the foregoing functionalities were readily available in it. Fermented feed can ameliorate the overall nutritional quality of fodder as well as animal growth and development performance (Azrinnahar et al., 2021, Linh et al., 2021 Yan et al., 2019). Furthermore, mannan oligosaccharides (MOS) and fructooligosaccharides (FOS) from yeast cell walls suppress enteric pathogens. Fermentation also increases the crude protein content while decreasing those of crude fiber and antinutritional factors inhibiting the secretion of various exogenous enzymes. Liza et al. (2022) reported that fermentation induces amylase and protease that degrade complex carbohydrates and proteins into simpler nutrient forms that are readily accessible energy sources. The observed increases in the growth performance of turkey broilers supplemented with β-glucan and FR indicate may be attributed to the aforementioned factors.

Here, β-glucan and FR supplementation significantly affected carcass, breast, and thigh weight possibly because β-glucan stimulated growth and/or FR increased the crude protein content. β-glucan (Jayachandran et al., 2018) and FR (Azrinnahar et al., 2021) increase the activity of the gastrointestinal tract and its microbiota, thereby accelerating muscle protein synthesis and, by extension, increasing breast and thigh weight. β-glucan also prevents the adhesion of pathogenic bacteria in the gut. Hence, it may have lowered the competition for nutrients between the pathogens and the chicks and increased the efficiency with which the latter utilize nutrients to build muscle mass. However, β-glucan supplementation had no apparent effect on the weights of the carcass and the internal organs, excepting liver and gizzard weight. Kovitvadhi et al. (2019) and Amer et al. (2023) also stated that β-glucan supplementation did not alter the carcass characteristics, the dressing percentage, or the weights of guts, or gizzards. Amer et al. (2023) also demonstrated that the energy latent in β-glucan was not applied toward muscle formation, thus did not affect dressing percentage. However, the supplement of β-glucan or even FR increased weight of liver and gizzard, it might be related to nutrient digestibility and immunity of turkeys. The results reported no adverse effects of β-glucan supplementation on turkey meat pH or cooking loss. Meat water content characteristics are important to consumers. Nevertheless, the meat water content did not significantly differ among treatment groups here. Even though the FR treatment resulted in nonsignificantly lower breast muscle cooking loss than the other treatments, FR supplementation did not affect the meat water content or holding capacity (Kovitvadhi et al., 2019, Moon et al., 2016). Zhang et al. (2020) showed that β-glucan supplementation reduced water loss during cooking. This effect is important as juiciness increases with decreasing cooking loss, and this quality factor is essential to customer satisfaction. Future research on meat quality should aim to clarify the mechanisms accounting for this apparent discrepancy.

FA composition also contributes to meat flavor. Elevated saturated and monounsaturated fatty acid content results in high tenderness, juiciness, flavor, and overall satisfaction scores. In contrast, high polyunsaturated fatty acid content renders the carcass fat soft and acidic and imparts an undesirable odor and texture to the meat. Little is known about the effects of *S. cerevisiae-*fermented rice and β-glucans on the FA content of broiler muscle tissue. The expected results should be the significant increase in MUFA and PUFA in breast muscle. However, FR and GLU in our study did not have that function. The increased polyunsaturated fatty acid content recorded in chicken broiler meat without statistically significant differences. Amer et al. (2023) stated that chicken meat processing and storage are affected by its chemistry. Amer et al. (2023) indicated that dietary β-glucans might affect lipid metabolism. The metabolites generated during fermentation and the yeast probiotics in fermented feed might positively influence the broiler meat FA content (Sun et al., 2022). Chen et al. (2019) reported similar findings for the muscle tissues of swine that were fed fermented fodder. Yan et al. (2019) stated that lowering the linoleic acid and total polyunsaturated fatty acid content did not affect the stearic acid or palmitic acid content in muscle tissue. The free FA content in chicken meat increases with dietary yeast content. The probiotics in fermented feed have beneficial to intestinal flora, therefore, inhibit PUFA oxidation (Benamirouche et al., 2020). Moon et al. (2016) mentioned that the stress of growing, transportation, pre-slaughter handling, and processing significantly affects meat quality. As dietary β-glucan can reduce oxidative stress in growing poultry, it should significantly influence meat quality. However, the amount of β-glucan adding in the diets of current study seems insufficient for reducing oxidative stress and affecting lipid metabolism. As mentioned in Amer et al. (2022), the supplement of β-glucan up to 120 mg Kg^−1^ affected blood profiles significantly, the lower amount did not record any effects. Nonetheless, numerous other factors also affect meat quality including diet composition, genetic structure, sex, housing management, slaughter method, and muscle fiber type (Lombardi-Boccia et al., 2005).

Blood parameters reflect the physiological, pathological, and nutritional status of animals. Alterations in hematological metrics can serve to determine the effects of dietary nutritional factors and additives on living organisms. Here, FR and β-glucan supplementation modulated the blood parameters and lowered cholesterol levels in broiler chickens; however, no statistically significant differences were observed. The results were in line with the study of Kovitvadhi et al. (2019), there were no significant records in blood chemistry including protein, albumin, cholesterol, triglyceride. Cao et al. (2017) demonstrated that β-glucan downregulates the genes controlling cholesterol, glycerolipid, and fatty acid biosynthesis as well as gluconeogenesis. β-glucan combines with bile acid, lowers blood cholesterol levels, increases gastrointestinal tract viscosity, delays gastric emptying and carbohydrate absorption, and lowers blood sugar levels (Amer et al., 2022). *Saccharomyces cerevisiae*-fermented rice and β-glucan induce digestive enzymes, improve nutrient digestibility, accelerate nutrient absorption, lengthen the intestinal villi, and increase the available amino acid content for plasma total protein and albumin biosynthesis (Abd-Elsamee et al., 2021). Psomas et al. (2003) found that *S. cerevisiae* removes intestinal cholesterol. In humans, β-glucan lowers serum cholesterol (Cicero et al., 2020). Hence, FR supplementation may also decrease blood cholesterol levels. The administration of yeast, *S. cerevisiae*, to recover bile acid increases the availability of free cholesterol as a bile acid precursor and, therefore, decreases serum cholesterol levels (Azrinnahar et al., 2021).

Immune organ indices and antibody titers are used to evaluate broiler immunity. Choi et al. (2014) found that the relative thymus, spleen, and bursa of Fabricius weights did not markedly differ among broilers given fermented feed. In contrast, we discovered that the broilers administered fermented feed presented significantly higher thymus indices than those fed the control diet. Ding et al. (2017) reported that animals given β-glucan supplementation exhibited higher organ indices than those administered a normal diet. Jacob and Pescatore (2017) showed that β-glucan stimulated precursor cell production in bone marrow, thereby increasing immunocyte migration into the lymphoid organs. In broilers, the immune response to fermented feed supplementation varies with the lymphoid compartment as each organ has its unique structure and function. Alterations in thymic mass may be associated with changes in lymphoid organ function. Thus, thymus indices might increase in response to recurrent or severe infection and a resultant diminished capacity to sustain production potential (Fasina et al., 2006). According to Ahmad et al. (2012), the immune response altered the relative mass of the thymus and the bursa of Fabricius. Bacterial cell walls also contain β-glucan which binds proteins and immune cells and, by extension, activates the immune response and/or increases antibody titers in birds. The observed increases in antibody titer against Newcastle disease virus in response to β-glucan and FR supplementation may be explained by the fact that fermented feed significantly improved cellular immunity parameters, namely, the serum interleukin (IL)-1, IL-2, and tumor necrosis factor (TNF)-α levels and the concanavalin A (Con A) stimulation index in the chicks. Fermented feed supplementation in chicks enhanced their humoral immunity as they displayed elevated serum IL-4 and IL-6 levels and B-lymphocyte proliferation (Zhu et al., 2020). β-glucan induces an immune response by binding complement receptor type three (CR3), Dectin-1, or Toll-like receptors (TLR) on neutrophils, macrophages, natural killer (NK) cells, and dendritic cells (DC) (Walachowski et al., 2017). Moreover, dietary *S. cerevisiae* increases immunoglobulin A (IgA) secretion from the intestinal mucosae as well as the cell-mediated and humoral immune responses (Kiarie et al., 2019).

## 5 Conclusion

The integration of 4% β-glucan and 4% *S. cerevisiae*-fermented rice improved the growth performance of turkey broilers by reducing their feed conversion ratios. β-glucan and fermented rice supplementation ameliorated the carcass characteristics without contributing to cooking loss or altering the pH of the breast and thigh meat. The foregoing treatments did not modulate the chemical composition, fatty acid profile, or quality of the meat. Future investigations should determine the significance of the fact that the total cholesterol levels were lower in the birds administered the fermented rice and the β-glucan than they were in those on the control diet. The present study showed that 4% β-glucan and 4% fermented rice supplementation enhanced the immune response to Newcastle disease virus in 28-day and 42-day turkey broilers, respectively. It could be recommended that 4% β-glucan and 4% *S. cerevisiae*-fermented rice could widely applied for turkey performance and health. The further investigation of fermented rice and β-glucan should be implemented in other poultry species.

## Declaration of Competing Interest

The authors declare that they have no known competing financial interests or personal relationships that could have appeared to influence the work reported in this paper.
